# Siderophore for Lanthanide and Iron Uptake for Methylotrophy and Plant Growth Promotion in *Methylobacterium aquaticum* Strain 22A

**DOI:** 10.3389/fmicb.2022.921635

**Published:** 2022-07-07

**Authors:** Patrick Otieno Juma, Yoshiko Fujitani, Ola Alessa, Tokitaka Oyama, Hiroya Yurimoto, Yasuyoshi Sakai, Akio Tani

**Affiliations:** ^1^Institute of Plant Science and Resources, Okayama University, Okayama, Japan; ^2^Graduate School of Science, Kyoto University, Kyoto, Japan; ^3^Graduate School of Agriculture, Kyoto University, Kyoto, Japan

**Keywords:** *Methylobacterium* species, lanthanide, lanthanophore, siderophore, plant growth promoter, heavy metal sequestration

## Abstract

*Methylobacterium* and *Methylorubrum* species are facultative methylotrophic bacteria that are abundant in the plant phyllosphere. They have two methanol dehydrogenases, MxaF and XoxF, which are dependent on either calcium or lanthanides (Lns), respectively. Lns exist as insoluble minerals in nature, and their solubilization and uptake require a siderophore-like substance (lanthanophore). *Methylobacterium* species have also been identified as plant growth-promoting bacteria although the actual mechanism has not been well-investigated. This study aimed to reveal the roles of siderophore in *Methylobacterium aquaticum* strain 22A in Ln uptake, bacterial physiology, and plant growth promotion. The strain 22A genome contains an eight-gene cluster encoding the staphyloferrin B-like (sbn) siderophore. We demonstrate that the sbn siderophore gene cluster is necessary for growth under low iron conditions and was complemented by supplementation with citrate or spent medium of the wild type or other strains of the genera. The siderophore exhibited adaptive features, including tolerance to oxidative and nitrosative stress, biofilm formation, and heavy metal sequestration. The contribution of the siderophore to plant growth was shown by the repressive growth of duckweed treated with siderophore mutant under iron-limited conditions; however, the siderophore was dispensable for strain 22A to colonize the phyllosphere. Importantly, the siderophore mutant could not grow on methanol, but the siderophore could solubilize insoluble Ln oxide, suggesting its critical role in methylotrophy. We also identified TonB-dependent receptors (TBDRs) for the siderophore–iron complex, iron citrate, and Ln, among 12 TBDRs in strain 22A. Analysis of the siderophore synthesis gene clusters and TBDR genes in *Methylobacterium* genomes revealed the existence of diverse types of siderophores and TBDRs. *Methylorubrum* species have an exclusive TBDR for Ln uptake that has been identified as LutH. Collectively, the results of this study provide insight into the importance of the sbn siderophore in Ln chelation, bacterial physiology, and the diversity of siderophore and TBDRs in *Methylobacterium* species.

## Introduction

*Methylobacterium* and *Methylorubrum* species are pink-pigmented bacteria capable of utilizing a variety of C1 compounds (Vuilleumier et al., 2009). These bacteria can colonize a wide range of environments and are among the most abundant bacterial genera associated with the plant phyllosphere (Knief et al., 2010). Their phyllospheric abundance can be attributed to their methylotrophy and the presence of methanol, which is produced mainly as a byproduct of plant pectin metabolism during cell wall synthesis (Fall and Benson, 1996). The contribution of methanol to the colonization was demonstrated by the reduced colonization capability of methylotrophy-deficient *Methylorubrum extorquens* strain AM1 mutants (Sy et al., 2005). Therefore, methylotrophy provides a selective advantage to the genus over other bacterial genera in the phyllosphere. Furthermore, the phyllosphere colonization by *Methylobacterium* species has been shown to vary with plant species, session, and growth stage (Omer et al., 2004). *Methylobacterium* species have also been identified as plant growth-promoting bacteria (PGPB) (Tani et al., 2015; Yurimoto et al., 2021) that enhance plant abiotic stress tolerance (Kumar et al., 2019).

*Methylobacterium* species have been extensively studied for methylotrophy in Gram-negative bacteria. Methanol is oxidized to formaldehyde by pyrroloquinoline quinone (PQQ)-dependent methanol dehydrogenases (MDHs) (Anthony, 2004). Generally, MDHs are classified into two types, MxaF and XoxF, which utilize calcium and lanthanide (Ln) as cofactors, respectively (Keltjens et al., 2014). Ln acts as the transcriptional activator that induces the XoxF-MDH while repressing MxaF-MDH (Haque et al., 2015). XoxF-MDH is more commonly conserved among *Methylobacterium* species than MxaF-MDH, projecting Ln as an integral mineral for methylotrophy (Alessa et al., 2021).

In nature, Ln occurs as mineral rocks that solubilize in sub-nanomolar concentration (Brown et al., 1990). Ln must be transported to the cytosol for Ln-dependent methanol metabolism to occur. A total of 10 genes, including TonB-dependent receptor (TBDR) and ATP-binding cassette (ABC)-type transporter analogous to the siderophore-mediated iron transport system, have been identified in the trafficking of Ln in *M. extorquens* strain AM1 (Roszczenko-Jasińska et al., 2020). Based on the inevitable need to enhance the bioavailability of Ln and the similarity of Ln-iron uptake systems, an Ln chelator termed lanthanophore has been fronted. Recently, gene candidates for lanthanophore biosynthesis [lanthanide chelation cluster (LCC)] were identified in strain AM1. The LCC cluster resembled the genes for aerobactin synthesized in *Grimontia hollisae*. However, the LCC cluster does not encode a complete gene set for aerobactin synthesis. Therefore, the product of LCC lanthanophore should be chemically and structurally different from aerobactin, although the chemically synthesized aerobactin siderophore was shown to bind Ln (Zytnick et al., 2022).

Bacteria rely on siderophores to acquire iron. Siderophores are low-molecular-weight compounds (200–2,000 Da) with a high affinity for iron (Ahmed and Holmström, 2014). They are structurally diverse and classified into three main families based on characteristic functional groups hydroxamate, catecholate, and hydroxycarboxylate (Sah and Singh, 2015). Siderophores also form stable complexes with other metal cations (Neubauer et al., 2000), sequester heavy metals (Chaturvedi et al., 2012), regulate oxidative stress (Adler et al., 2012), and provide antibacterial activity (Braun et al., 2009). Once iron is chelated, the complex is transported through a TBDR utilizing energy that is derived from the proton motive force and transmitted from the TonB-ExbB-ExbD proteins (Noinaj et al., 2010). TBDRs have also been reported to transport vitamin B12, saccharides, and aromatic compounds (Fujita et al., 2019).

Siderophore production is one of the important bacterial functions in plant–bacteria and bacteria–bacteria interaction (Kramer et al., 2021); however, it has not been characterized well in *Methylobacterium* species and few strains in the genus have been reported to synthesize siderophores (Lacava et al., 2008). The recent finding of the aerobactin-like LCC gene cluster in strain AM1 showed that siderophores can play a critical role in Ln acquisition, which leads to the induction and activation of a novel enzyme, Ln-dependent XoxF-type MDHs. The mechanism for Ln-dependent expression switching (Ln-switch) between Ca^2+^-dependent MxaF and Ln-dependent XoxF is an important research topic in bacterial methylotrophy, and the elucidation of the siderophore function as lanthanophore is crucial to dissecting the molecular mechanism of Ln-switch.

The genera *Methylobacterium* and *Methylorubrum* are diverse, comprising 63 species and categorized into three major clades, A, B, and C. Clade C has a different methylotrophy gene repertoire, including a lack of *mxaF* in some strains, signifying the importance of the uptake of Ln in this group. This clade also has a different set of genes for C1 compound metabolism, such as the glutathione-dependent formaldehyde dehydrogenase pathway (Yanpirat et al., 2020), and is characterized by a high GC-rich genome (Alessa et al., 2021). *Methylobacterium aquaticum* strain 22A was isolated from *Racomitrium japonicum* (Tani et al., 2012). The strain belongs to clade C and its complete genome information is available (Tani et al., 2016). Strain 22A has plant growth promotion capability, although the mechanism has not yet been established. Siderophore is proposed to contribute to the plant growth promotion effect. In this paper, we investigate the function of the staphyloferrin B-like siderophore from strain 22A in plant growth promotion, bacterial physiology, and its involvement in methylotrophy. Furthermore, we aim to enhance knowledge on siderophore synthesis and receptors, using the genome information of all type strains of *Methylobacterium* and *Methylorubrum* species (Alessa et al., 2021).

## Results

### Gene Organization of Siderophore Synthesis Cluster in Strain 22A Genome

The strain 22A genome contains a siderophore synthesis cluster composed of eight genes (Maq22A_c25425 to Maq22A_c25465) with a similarity of 12% to the staphylobactin cluster (BGC0000943) according to anti-SMASH analysis. The strain 22A siderophore cluster, herein named *sbnA-H*, also showed 33–56% identity to a well-characterized hydroxycarboxylate-type staphyloferrin B cluster (*sbnA-I*) from *S. aureus* (Supplementary Table 1 and Supplementary Figure 1) (Cheung et al., 2009) and *Ralstonia solanacearum* (Bhatt and Denny, 2004). The N- and C-terminal portions of SbnF of strain 22A showed 34 and 33% similarity to *sbnE* and *sbnF* of *S. aureus*, respectively, suggesting that *sbnE* and *sbnF* are fused in strain 22A. Similar gene clusters were also found in six other *Methylobacterium* type strains in a non-taxonomy-specific manner. We hypothesized that the strain 22A *sbn* cluster synthesizes a staphyloferrin B-like siderophore.

Staphyloferrin B is biosynthesized by the SbnA-SbnI encoded by *sbn* gene cluster in *Staphylococcus aureus* (Cheung et al., 2009). SbnA, SbnB, and SbnG generate L-diaminopropionic acid (L-DAP), α-ketoglutarate (α-KG), and citrate, respectively, as the siderophore precursors. SbnE condenses citrate and L-DAP to citryl-DAP. SbnF condenses citryl-1,2-diaminoethane (DAE) and L-DAP to L-DAP-citryl-DAE. SbnC catalyzes the amide linking of the precursor and α-KG to produce staphyloferrin B (Cheung et al., 2009). The *sbn* cluster is also proposed to synthesize a non-characterized siderophore staphylobactin, which is larger than staphyloferrin A or B by >300 Da (Dale et al., 2004).

### Characterization of Siderophore Mutants of Strain 22A

The spent medium of strain 22A was negative for catecholate and hydroxamate siderophore tests but positive for hydroxycarboxylate-type siderophore (Supplementary Figure 2A). Knockout mutants for *sbnC* and *sbnF* that were annotated as *iucA/iucC* family siderophore biosynthesis proteins were constructed to generate Δ*sbnC*, Δ*sbnF*, and Δ*sbnCF*. Gene disruptants were also generated for the other individual *sbn* genes and a hypothetical gene (Maq22A_c25465) at the distal end of the cluster. Strain 22A wild type showed a positive reaction on chrome azurol S (CAS) agar plate, which is indicative of siderophore production whereas Δ*sbnA*, Δ*sbnD*, Δ*sbnF*, Δ*sbnH*, and Δ*sbnCF* were negative (Supplementary Figure 2B).

The strain 22A *sbn* gene cluster contains two of the three *iucA/iucC* family genes (*sbnC* and *sbnF*) involved in many general non-ribosomal peptide synthetase (NRPS)-independent siderophore (NIS) biosynthesis. NIS biosynthesis utilizes at least one of these enzymes for siderophore synthesis as a crucial component (Challis, 2005). Due to this important potential role, we used Δ*sbnCF* as a representative siderophore mutant throughout this study.

**FIGURE 1:**
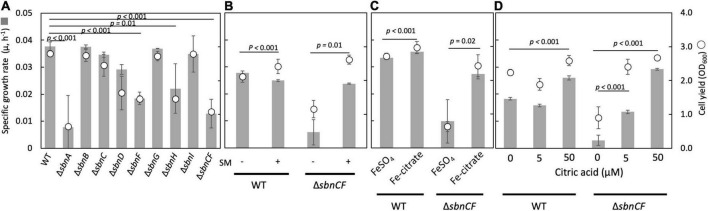
**(A)** Growth of *M. aquaticum* strain 22A wild type (WT), siderophore *sbn* gene mutants on succinate with FeSO_4_. **(B)** Growth of WT and Δ*sbnCF* on succinate with/without the addition of a spent medium (SM) of the WT. **(C)** Growth of WT and Δ*sbnCF* on succinate in the presence of 100 μM FeSO_4_ or 100 μM iron citrate. **(D)** Growth of WT and Δ*sbnCF* on succinate in the presence of 50 μM FeSO_4_ and different concentrations of citrate. Bars, specific growth rate (μ, h^–1^); circles, cell yield (OD_600_). All results are presented as the average ± standard deviation (SD) (biological triplicates).

The Δ*sbnC* showed a reduced CAS reaction and a normal growth, whereas Δ*sbnA, ΔsbnD, ΔsbnA, ΔsbnD*, and Δ*sbnCF* showed slower growth compared to the wild type, on succinate in the presence of 100 μM FeSO_4_ (Figure 1A). The wild-type spent medium complemented the growth of Δ*sbnCF* (Figure 1B), suggesting the inability of the siderophore synthesis of Δ*sbnCF*. Interestingly, Δ*sbnCF* was able to grow on succinate in the presence of iron citrate (>50 μM) (Figure 1C). Furthermore, the combined supplementation of FeSO_4_ (50 μM) and citrate rescued Δ*sbnCF* in a citrate concentration-dependent manner (Figure 1D). These results suggested that although the sbn siderophore contributes to the utilization of FeSO_4_, strain 22A has another mechanism to acquire iron citrate.

**FIGURE 2:**
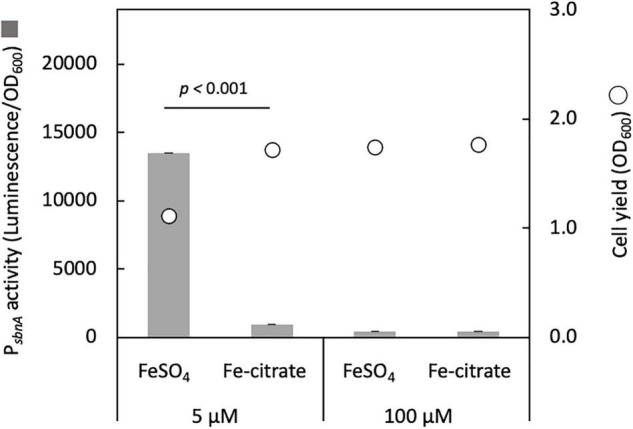
*sbnA* promoter activity assay with luciferase as a reporter in the wild type grown on succinate in the presence of 5 and 100 μM FeSO_4_ or iron citrate. Bars, *sbnA* promoter activity (arbitrary unit, luminescence/OD_600_); circles, cell yield (OD_600_). The results were presented as the average ± standard deviation (SD) (biological triplicates).

We cloned the promoter region of *sbnA* (P*_*sbnA*_*, 644 bp) into pAT06, a promoter-reporter vector containing bacterial luciferase genes as a reporter and introduced it into strain 22A wild type. P*_*sbnA*_* showed high activity with 5 μM FeSO_4_ and low activity with 100 μM FeSO_4_ or the presence of iron citrate (Figure 2). This result suggested that *sbn* expression is induced by low iron availability and repressed by iron citrate.

### Siderophore Mediates the Interaction With Plants

**FIGURE 3:**
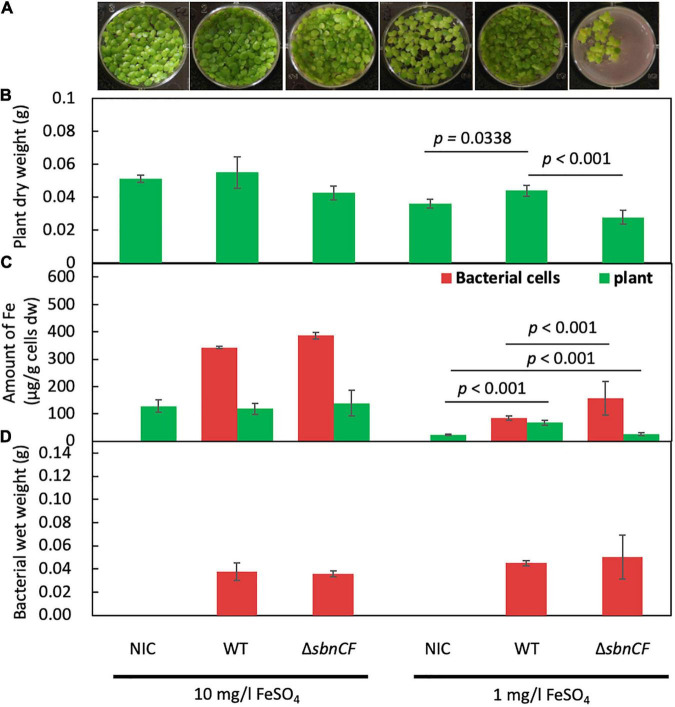
**(A)** Growth of duckweed plants in liquid 1/2 MS medium inoculated with none (non-inoculated control, NIC), strain 22A wild type (WT), and Δ*sbnCF*, under 10 and 1 mg/l FeSO_4_ for 28 days. Representative pictures are shown for each treatment. **(B)** Dry weight of plants after cultivation. **(C)** Iron content in the plants and bacterial cells. **(D)** Bacterial mass measured as wet weight after cultivation. All the data are reported as the mean of three replicates ± standard deviation (SD).

Although strain 22A has been identified as a potential PGPB in field and pot conditions (Tani et al., 2015), the growth promotion mechanism has not yet been studied. We assessed the contribution of siderophore to this plant–bacterial interaction. We used duckweed (*Lemna gibba* p8L) as a plant model. Strain 22A wild type and Δ*sbnCF* were inoculated into 1/2 Murashige–Skoog (MS) medium under two iron regimes. The wild type and Δ*sbnCF* showed no plant growth promotion under sufficient iron conditions (10 mg/l) (Figures 3A,B). In iron-limited conditions (1 mg/l), the wild-type strain 22A promoted duckweed growth, whereas Δ*sbnCF* suppressed the plant growth. These results suggested that the growth promotion effect is dependent on iron availability, and the siderophore is directly involved in its mechanism.

The treated plants were subjected to iron content measurement with inductively coupled plasma mass spectrometry (ICP-MS). The plants in sufficient iron conditions contained almost constant iron levels irrespective of the genotypes of strain 22A (Figure 3C), suggesting that there was no competition for iron between the plants and the bacteria. In iron-limited conditions where the plants showed relatively smaller biomass, the plant iron content was increased by the presence of the wild type, suggesting that strain 22A assisted in iron acquisition in the plants. In the presence of Δ*sbnCF*, the plants contained lower iron compared to the treatment with the wild type, and the Δ*sbnCF* cells contained more iron than the wild type. These results suggested that the mutant competed with the plant for iron. Meanwhile, the bacterial cell mass assessed as wet weight in these conditions showed no difference between the wild type and Δ*sbnCF* (Figure 3D).

The growth promotion effect by the wild type was also reproduced in *Arabidopsis thaliana*, under low iron conditions (1 mg/l) (Supplementary Figure 3A), suggesting that the iron dependency of the growth promotion effect is not dependent on plant species. *A. thaliana* was inoculated with a rifampicin-resistant strain 22A (22A-rif) and a kanamycin-resistant Δ*sbnCF* (Δ*sbnCF-*km) individually or in co-inoculation under 1 mg/l FeSO_4_ (Supplementary Figure 3B). Δ*sbnCF*-km colonized the plant phyllosphere better than 22A-rif in both conditions. These results suggested that Δ*sbnCF* could acquire iron in the *A. thaliana* phyllosphere without the siderophore.

### Sbn Siderophore Is Involved in Ln Uptake and Ln-Dependent Methylotrophy

**FIGURE 4:**
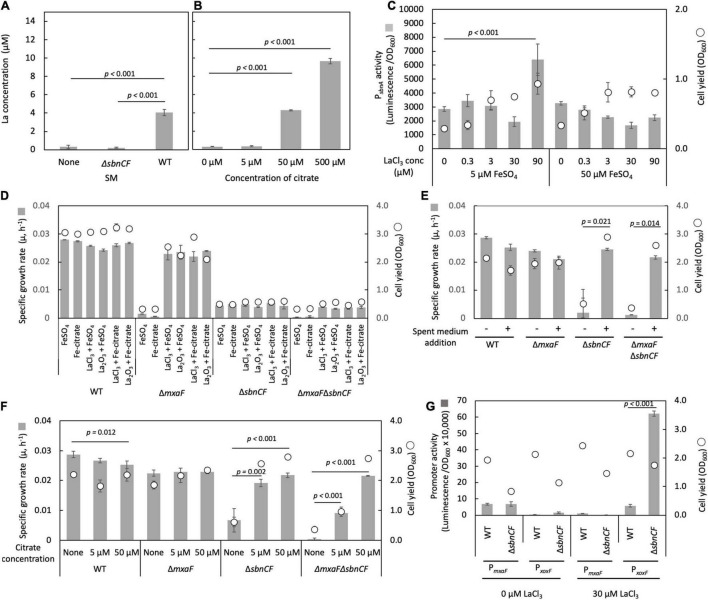
**(A)** Solubilization of La_2_O_3_ by the spent media (SM) of the wild-type strain 22A (WT) and Δ*sbnCF*, and non-inoculated medium control. **(B)** Solubilization of La_2_O_3_ by citrate (5–500 μM). **(C)** P*_*sbnA*_* activity in Δ*mxaF* in the presence of varied concentrations of LaCl_3_ under 5 and 50 μM FeSO_4_. **(D)** Growth of the WT, Δ*mxaF*, Δ*sbnCF*, and Δ*mxaF*Δ*sbnCF* on methanol in the presence of 100 μM FeSO_4_, 100 μM iron citrate, 9 mg/l La_2_O_3_, and 30 μM LaCl_3_, and their mixtures. **(E)** Growth of the same set of mutants on methanol in the presence/absence of the spent medium of the WT, under 100 μM FeSO_4_ and 30 μM LaCl_3_. **(F)** Growth of the same set of mutants on methanol in the presence of different concentrations of citrate, under 50 μM FeSO_4_ and 30 μM LaCl_3_. **(G)** P*_*mxaF*_* and P*_*xoxF*_* activity in WT and Δ*sbnCF* grown on methanol plus succinate in the absence/presence of 30 μM LaCl_3_. All data represent the mean of three replicates ± standard deviation (SD).

We tested whether the spent medium of the wild type can solubilize insoluble Ln. La_2_O_3_ was suspended in the spent medium of the wild type and Δ*sbnCF*, followed by incubation for 24 h and quantification of soluble La. We could detect a 24-folds higher amount of La in the wild-type spent medium compared to the non-inoculated medium and Δ*sbnCF* spent medium (Figure 4A), suggesting that sbn siderophore is involved in Ln solubilization and chelation. We also confirmed that >50 μM citrate solubilized La_2_O_3_ (Figure 4B).

Δ*mxaF* can grow on methanol only in the presence of La^3+^, due to intact XoxF (Masuda et al., 2018). Δ*mxaF* containing the P*_*sbnA*_* reporter vector was cultivated on succinate plus methanol under various concentrations of LaCl_3_ and FeSO_4_. The promoter activity was the control level in LaCl_3_ concentration in the range of 0.3–30 μM under both 5 and 50 μM FeSO_4_ (Figure 4C). A high concentration of LaCl_3_ (90 μM) activated P*_*sbnA*_* under 5 μM but not under 50 μM FeSO_4_, suggesting that LaCl_3_ created iron scarcity, increasing the need for siderophore synthesis.

To further examine the involvement of siderophore in Ln solubilization, we generated Δ*mxaF*Δ*sbnCF*. The wild type grew on methanol utilizing FeSO_4_ or iron citrate and either forms of La (LaCl_3_ or La_2_O_3_) (Figure 4D). Without the addition of an iron source, the wild type could not grow, however, La source omission did not affect its growth (data not shown). Δ*mxaF* growth on methanol was dependent on either of the La forms and iron. Δ*sbnCF* and Δ*mxaF*Δ*sbnCF* could not grow on methanol in all conditions, even under a high concentration of iron citrate (100 μM) that could complement their growth on succinate (Figure 1C). We also found that Δ*mxaF*Δ*sbnCF* was able to grow on methanol in the presence of LaCl_3_ and the wild-type spent media (Figure 4E) or with citrate (but not iron citrate) in a concentration-dependent manner (Figure 4F). These results suggested that siderophore is not required for methylotrophic growth in the presence of citrate, but that it serves as an Ln chelator in the absence of citrate, and that citrate also chelates Ln for uptake.

We assessed the promoter activity of MDH genes (*xoxF* and *mxaF)* in the wild type and Δ*sbnCF* grown on methanol plus succinate in the absence/presence of 30 μM LaCl_3_. The wild type showed a clear Ln-dependent switch of MDH gene expression (Figure 4G). In the presence of LaCl_3_, Δ*sbnCF* showed more than 10 times higher activity of P*_*xoxF*_* than the wild type. Judging from the difference in final Δ*sbnCF* cell yield between 0 and 30 μM LaCl_3_, Δ*sbnCF* has methanol oxidation capacity in the presence of LaCl_3_, which means that La^3+^ uptake occurs by an unknown mechanism to activate XoxF. The reason for the high activity of P*_*xoxF*_* is unclear, but it is possible that sbn siderophore is involved in the maintenance of La^3+^ concentration in the cells, and the resultant high La^3+^ content in the cells resulted in high induction of *xoxF*.

### Role of Sbn Siderophore in Biofilm Formation, Resistance to Reactive Oxygen Species, and Heavy Metals

The biofilm formation was inversely proportional to the medium iron concentration in the wild type, whereas supplementation with iron citrate resulted in biofilm formation in a concentration-dependent manner in Δ*sbnCF* (Supplementary Figure 4A). This result suggests that iron availability and siderophore production can influence biofilm formation in strain 22A. We could not compare biofilm formation without iron citrate since Δ*sbnCF* could not grow.

Δ*sbnCF* was more susceptible to hydrogen peroxide (1 mM) and sodium nitrite (10 mM) but not to diamide (10 mM) compared to the wild type (Supplementary Figure 4B), suggesting that siderophore synthesis is involved in resistance to the oxidative stress.

**FIGURE 5:**
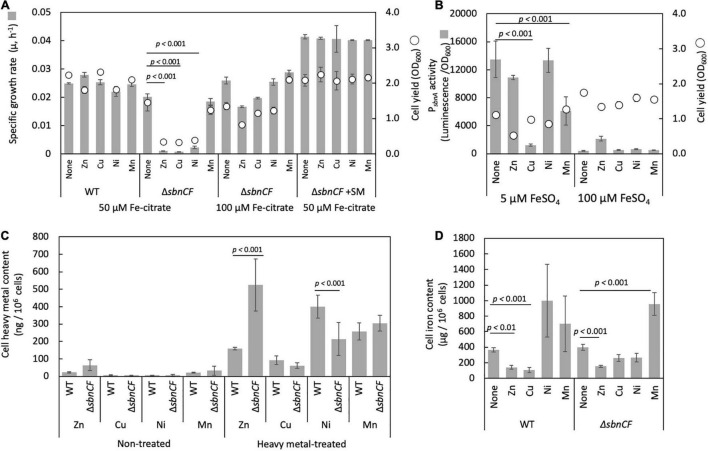
**(A)** Growth of the wild-type strain 22A and Δ*sbnCF* on succinate in the presence of 100 μM of ZnSO_4_, CuSO_4_, NiSO_4_, and MnSO_4_ under 50 μM iron citrate, growth of Δ*sbnCF* under 50 μM under 100 μM iron citrate. Growth of Δ*sbnCF* on succinate in the presence of 100 μM heavy metals under 50 μM iron citrate supplemented with 22A wild-type spent media. **(B)** P_sbnA_ activity in the wild-type strain 22A in the presence of 100 μM of each heavy metal under 5 and 100 μM FeSO_4_. **(C)** Heavy metal accumulation in the wild-type strain 22A and Δ*sbnCF* cells supplemented with 25 μM heavy metals (HM) or without (NT). **(D)** Iron accumulation in the cells of wild-type strain 22A and Δ*sbnCF* grown on succinate in the presence of 25 μM of ZnSO_4_, CuSO_4_, NiSO_4_, and MnSO_4_, under 100 μM iron citrate. Data represent the mean of three replicates ± standard deviation (SD).

The growth of strain 22A and Δ*sbnCF* in the presence of 50 μM iron citrate was not affected by 10 μM ZnSO_4_, CuSO_4_, NiSO_4_, and MnSO_4_ (data not shown). The growth of Δ*sbnCF* was inhibited by 100 μM ZnSO_4_, CuSO_4_, and NiSO_4_, but no significant inhibition was seen on the wild-type strain (Figure 5A). Supplementation of wild-type spent media to Δ*sbnCF* restored its growth under heavy metals. Higher iron concentration (100 μM iron citrate) also alleviated heavy metal toxicity in Δ*sbnCF*. *sbnA* promoter activity in the wild type under 100 μM of each heavy metal and two iron concentration regimes was examined (Figure 5B). P*_*sbnA*_* showed a control-level activity under ZnSO_4_ and NiSO_4_, and repressed activity under CuSO_4_ and MnSO_4_ in the presence of 5 μM FeSO_4_. In the presence of 100 μM FeSO_4_, the promoter activity was generally repressed even in the presence of these metals, but not completely repressed in the presence of ZnSO_4_. The heavy metal content measurement revealed higher Zn accumulation and lower Ni accumulation in ZnSO_4_- and NiSO_4_-treated Δ*sbnCF* compared to the wild type, respectively (Figure 5C). The cellular iron content in the absence of heavy metals was comparable between 22A wild type and Δ*sbnCF* when they were grown in the presence of iron citrate (Figure 5D). The addition of ZnSO_4_ or CuSO_4_ reduced, whereas the addition of MnSO_4_ increased, iron accumulation in both strains. The addition of NiSO_4_ resulted in iron accumulation that was similar to that of the control in both the wild type and Δ*sbnCF*. Overall, these data suggested that sbn siderophore is involved in the heavy metal resistance of strain 22A; however, the mechanism of resistance for each metal may differ.

### Variety of Siderophore Biosynthesis Clusters in *Methylobacterium* Species

We found a total of 66 siderophore synthesis gene clusters among 60 genomes of *Methylobacterium* type strains and strain 22A through anti-SMASH analysis (Supplementary Table 2). They had varied homology to known siderophore gene clusters (7–100%) and could be classified into 10 groups based on similarity. A total of 13 species were associated with two or more siderophore synthesis clusters. Most of the siderophore biosynthesis gene clusters were composed of 6–16 genes (data not shown). We found that 41 type strains were positive in the CAS agar assay. Although no gene cluster was identified in nine strains, seven of them were positive in the CAS agar assay. The malleobactin-like siderophore gene cluster was dominantly found in clades A, A1, and A5. The taiwachelin-like siderophore gene cluster was found in clade A4 and some members in clade B. Clades B2, C1, and C2 members have no dominant siderophore gene cluster, and only three species have a staphylobactin-like siderophore gene cluster in clade C1, to which *M. aquaticum* belongs. A total of 15 other siderophore gene clusters had no similarity to known clusters. Thus, siderophore synthesis is a common characteristic among *Methylobacterium* species, although only some types of siderophore are distinctly conserved in phylogenetic clades.

Co-inoculation of 58 *Methylobacterium* type strains with strain 22A Δ*sbnCF* resulted in growth restoration by 21 strains. Many of the positive strains have taiwachelin (8 strains) or staphylobactin (6 strains), whereas strains containing malleobactin or ochrobactin were negative. Interestingly, only 62% of the strains with taiwachelin-like siderophore could enable iron deficiency recovery. All five strains with an *sbn* cluster could complement the growth of Δ*sbnCF. R. solanacearum* strain RS1000, which produces staphyloferrin B (Bhatt and Denny, 2004), was also able to rescue Δ*sbnCF*. The strains with an *sbn* cluster were all isolated from plants except for *M. ajmalii* strain IF7SW-B2T (Bijlani et al., 2021), implying the possible advantage of this type of siderophore in the plant-associated lifestyle.

### Characterization of TonB-Dependent Receptors in Strain 22A

The strain 22A genome encodes 12 TBDRs, among which, those for iron-siderophore complex, iron citrate, and possible Ln-lanthanophore should exist. We examined the phenotype of the gene disruption mutants or deletion mutants of these receptors in strain 22A. The 9 TBDR mutants not discussed here did not show any phenotypes in these assays.

**FIGURE 6:**
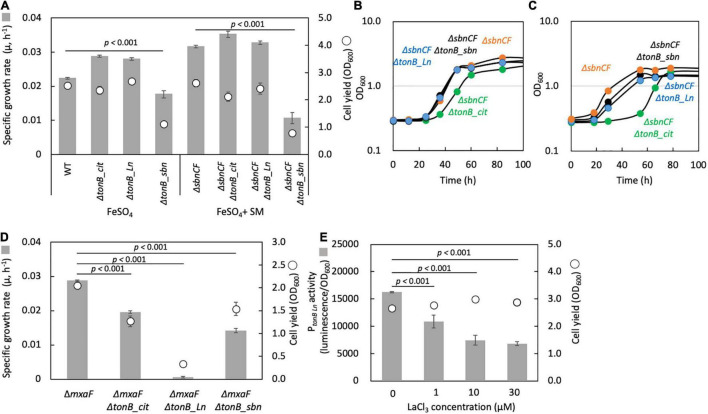
**(A)** Growth of the mutants of each TBDR gene generated under the wild-type strain 22A (WT) and Δ*SbnCF* backgrounds on succinate supplemented with 100 μM FeSO_4_ 10% WT spent medium (SM), respectively. **(B)** Growth of the mutants of each TBDR gene generated under the background of Δ*sbnCF* on succinate under 100 μM iron citrate. **(C)** Same experiment as **(B)** but with 50 μM citric acid and 50 μM FeSO_4_. **(D)** Growth of the mutants of each TBDR gene generated under the Δ*mxaF* background on methanol under 100 μM iron citrate and 30 μM LaCl_3_. **(E)** P*_*tonB_Ln*_* activity in WT grown on methanol in the presence of different concentrations of LaCl_3_. The gene disruption mutants were grown in the presence of kanamycin throughout the experiment. All data represent the mean of three replicates ± standard deviation (SD).

TonB-dependent receptor for sbn siderophore: A TBDR gene (Maq22A_c25420) was found in the upstream region of the *sbn* gene cluster and named *tonB_sbn*. Δ*tonB_sbn* showed slower growth with FeSO_4_, and Δ*sbnCF*Δ*tonB_sbn* could not grow on succinate with FeSO_4_ and wild-type spent medium (Figure 6A). Iron citrate supplementation rescued Δ*sbnCF*Δ*tonB_sbn* growth (Figure 6B). These results indicated that *tonB_sbn* is involved in the sbn-siderophore-iron complex transport.

TonB-dependent receptor for iron citrate: We found that a gene deletion mutant of Maq22A_c24885, herein named Δ*tonB_cit*, under a background of Δ*sbnCF*, showed a lag phase when grown on succinate with iron citrate (Figure 6B). Supplementation of 50 μM citrate and 50 μM FeSO_4_ also resulted in a lag phase (Figure 6C). *tonB_cit* is therefore involved in iron citrate transport. However, the mutant could still grow. There may thus be another TBDR that can transport iron citrate or one for iron chelated by other substances.

TonB-dependent receptor for Ln: A deletion mutant of Maq22A_c14845 (labeled as Δ*tonB_Ln*) under a Δ*mxaF* background did not grow on methanol with LaCl_3_ (Figure 6D). The promoter region of *tonB_Ln* was cloned into pAT06, and the promoter activity in the wild type showed lower expression in a higher LaCl_3_ concentration (Figure 6E). This suggests that *tonB_Ln* is involved in Ln transport, and its expression is repressed when Ln is well available.

### Variety and Distribution of TonB-Dependent Receptors in *Methylobacterium* Genomes

**FIGURE 7:**
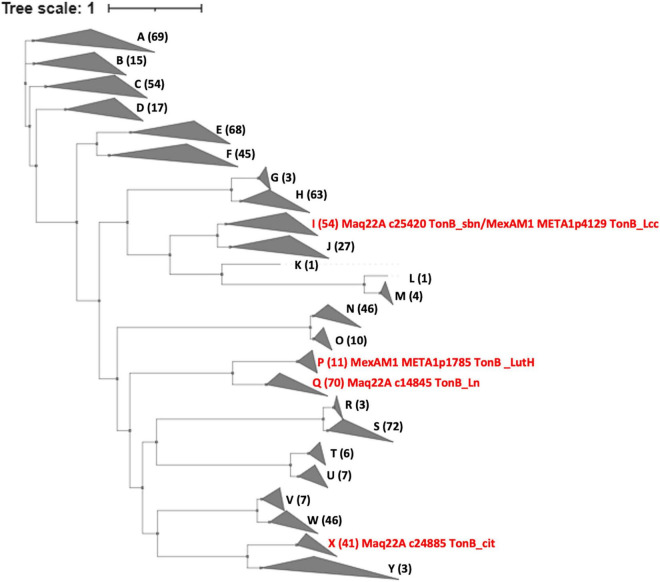
Phylogenetic tree and gene counts of TBDRs found in the genomes of different subclades of *Methylobacterium* type strains. The phylogenetic tree is constructed based on the amino acid sequences of TBDR genes. The *Methylobacterium* subclade definition is based on our previous report (Alessa et al., 2021). A total of 25 clades are labeled A-Y. The counts of TBDRs and description of characterized TBDRs are indicated in brackets. Details are presented in Supplementary Table 3.

Using the sequences of the TBDRs in strain 22A and the TBDRs found in *M. extorquens* strain AM1 (LutH, META1p1785, and TonB_Lcc, META1p4129) as queries, we searched TBDRs encoded in all type strains of *Methylobacterium* and *Methylorubrum* species (Figure 7 and Supplementary Table 3). TBDRs found in the genera were phylogenetically classified into 25 clusters, and each strain had 7–23 TBDRs in its genome. TonB_sbn is homologous to TonB_Lcc of strain AM1 and 52 other TBDRs. TonB_Ln is phylogenetically close to strain AM1 LutH and widely conserved with a total of 69 homologs in the genera; it is especially enriched in clades A4 and C1. LutH has only 10 homologs and is well conserved only in clade B (*Methylorubrum* species) and two clade A1 members. TonB_cit was also well conserved (40 homologous TBDRs), suggesting that the majority of *Methylobacterium* species can utilize iron citrate. TBDRs in strain 22A are phylogenetically diverse and each of them is widely conserved among *Methylobacterium* species.

## Discussion

The strain 22A *sbn* gene cluster showed 33–56% homology to *sbn* genes in *S. aureus* (Supplementary Table 1). The cluster lacks *sbnI*, whereas *sbnE* and *sbnF* are fused in strain 22A. We presumed that strain 22A siderophore has a similar structure to staphyloferrin B. Δ*sbnA, ΔsbnD, ΔsbnF, ΔsbnH*, and Δ*sbnCF* but not Δ*sbnC* showed defective growth on succinate under iron deficiency. Because SbnC catalyzes the critical final step for staphyloferrin B synthesis, the intermediate(s) produced without SbnC may still be active for iron chelation. SbnF in strain 22A (the fused SbnE and SbnF) may be more important to synthesize the intermediates. This point will be clarified when we determine the siderophore structure and the functions of each gene.

Δ*sbnCF* could not grow well with FeSO_4_ (Figure 1A), and its growth was complemented by the spent medium of the wild type or iron citrate (Figures 1B,C). *sbn* gene expression is induced by low iron availability and repressed by iron citrate (Figure 2). Citrate has a lower affinity (K_*d*_ < 0.260 nM) for iron than that of conventional siderophores (Fukushima et al., 2012) and acts as a general iron chelator for the bacterial community as opposed to individual siderophores (Mazoy et al., 1997). Therefore, strain 22A preferably utilizes iron citrate if available, without synthesizing sbn siderophore.

The sbn siderophore of strain 22A is one of the plant growth promotion factors (Figure 3). Bacterial siderophore supplementation abolishes iron deficiency in plants (Crowley et al., 1992). Interestingly, Δ*sbnCF* competed with plants for iron, which resulted in a low plant iron uptake. Because strain 22A can also utilize citrate as a carbon source, and citrate is one of the common organic acids in root exudates in response to iron deficiency (Ling et al., 2011; Yuan et al., 2015), we considered that Δ*sbnCF* overtook the iron complexed by plant-derived citrate. It should be noted that since iron citrate represses siderophore production, siderophore synthesis in the wild type precedes the plant citrate exudation, and the wild-type strain 22A did not become opportunistic. The growth promotion ability of strain 22A was also observed in *A. thaliana* in low iron conditions (Supplementary Figure 3). The siderophore was not essential for strain 22A to colonize the *A. thaliana* phyllosphere, but in return, the plant growth was repressed. Judging from its iron accumulation, Δ*sbnCF* can pirate iron citrate that is relatively abundant in the phyllosphere (Yokosho et al., 2009; Rellán-Álvarez et al., 2010). On the other hand, siderophore synthesis was a biological cost in the wild type.

We found that the sbn siderophore and citrate solubilize La_2_O_3_ (Figures 4A,B). The sbn siderophore was indispensable in the absence of citrate (Figures 4D,E) but was dispensable in the presence of citrate (not iron citrate) for XoxF- and Ln-dependent methylotrophic growth (Figure 4F). Thus, we concluded that the sbn siderophore is another type of “lanthanophore” involved in Ln uptake, in addition to the aerobactin-type siderophore in *M. extorquens* strain AM1 (Zytnick et al., 2022). Interestingly, sbn siderophore inactivation resulted in the overactivation of P*_*xoxF*_* in the presence of LaCl_3_ (Figure 4G). The expression of *xoxF* and *mxaF* is regulated by the Ln-switch; therefore, this *xoxF* overexpression indeed suppressed *mxaF*. It is generally accepted that siderophores play a role in the homeostasis of other metals other than iron. Most probably, the sbn siderophore plays a role not only in controlled Ln uptake but also in maintaining cellular Ln homeostasis. The siderophore-free La^3+^ in the periplasm might strongly induce XoxF through activation of MxbDM that is a two-component signaling system responsible for the expression of MDHs (Yanpirat et al., 2020). The exact reason for this high *xoxF* induction remains to be determined through investigation of the Ln-switch; however, here, we can add the sbn-type lanthanophore as one of the factors involved in the complex switching mechanism.

Iron concentration inversely impacted the biofilm formation in strain 22A wild type. Access to a sufficient amount of iron could deter biofilm formation since environmental stresses such as iron starvation generally trigger biofilm formation (Muhammad et al., 2020). This corroborated with other studies showing that iron depletion enhanced biofilm formation in different bacteria including *E. coli* (Hancock et al., 2008) and *Cupriavidus necator* (Li et al., 2019). In contrast, biofilm formation in Δ*sbnCF* was enhanced with an increasing iron concentration (Supplementary Figure 4A). Iron is essential for biofilm formation (Oliveira et al., 2017) and iron is a limiting growth factor for Δ*sbnCF* at the tested iron citrate concentrations (Supplementary Figure 4A). The enhanced biofilm formation in Δ*sbnCF* by iron citrate suggested that iron acquisition rather than sbn siderophore *per se* was the important determinant.

Δ*sbnCF* showed less tolerance to hydrogen peroxide and sodium nitrite (Supplementary Figure 4B), implying that this iron uptake system confers tolerance to oxidative stress. Iron is a dangerous metal due to its capacity to generate reactive oxygen species (ROS) through the Fenton reaction (Corpas et al., 2015). Therefore, iron homeostasis could be related to oxidative stress (Cornelis et al., 2011). *SirA*, a staphyloferrin B transport system, influences tolerance to ROS in *S. aureus* (Nobre and Saraiva, 2014). Whereas hydrogen peroxide increases intracellular hydroxyl radicals that lead to direct oxidation, diamide causes indirect oxidative stress by oxidizing glutathione (GSH) and furthering redox imbalance (Toledano et al., 2003). Therefore, iron mitigation by *sbn* siderophore could be ineffective for such an indirect oxidative stress mechanism.

The sbn siderophore of strain 22A contributes to heavy metal resistance, and the results of the cellular metal accumulation and P*_*sbnA*_* activity analyses indicated that toxicity and siderophore-mediated resistance mechanisms differ depending on the metal species (Figure 5). The heavy metal toxicity was also influenced by the iron concentration. This suggested possibility of uptake competition among the metals. ZnSO_4_ treatment did not have an effect on P*_*sbnA*_* activity, caused a high accumulation of Zn^2+^ in Δ*sbnCF*, and caused iron scarcity. The scarcity suggested that the sbn siderophore binds Zn^2+^, and hence, the siderophore prevents uncontrolled entry of Zn^2+^ into the cells (Braud et al., 2009). CuSO_4_ treatment repressed P*_*sbnA*_* activity, caused unchanged Cu^2+^ accumulation in the wild type and Δ*sbnCF*, and decreased iron content in the wild type. Therefore, copper susceptibility of the mutant may include iron scarcity caused by P*_*sbnA*_* repression but not Cu^2+^ accumulation. NiSO_4_ treatment did not have an effect on P*_*sbnA*_* activity and caused a higher accumulation of Ni^2+^ in the wild type. Thus, Ni susceptibility of the mutant was not caused by Ni accumulation but rather by repressed iron transport that is necessary to overcome Ni toxicity. MnSO_4_ treatment at the tested concentration did not cause any growth inhibition in the wild type and Δ*sbnCF*, suggesting that the sbn siderophore does not take part in the resistance. However, it caused decreased P*_*sbnA*_* activity, higher Mn, and iron accumulation in both cells. This contradictory decreased P*_*sbnA*_* activity and higher iron accumulation could not be explained well only with these data. These metals are essential mineral elements that exhibit important interactions and possible competitive inhibition of transport. The biochemical resistance mechanism in which the sbn siderophore participates awaits further characterization of the purified siderophore including its binding capacity and identification of the transporters for each metal.

Siderophore production is a common characteristic in *Methylobacterium* species bearing similarity and disparity among groups of individual strains (Supplementary Table 2). Malleobactin- and taiwachelin-type siderophore gene clusters may have been conserved through their evolution and phylogeny whereas the horizontal acquisition of the other siderophore gene clusters is dependent on their habitat as opposed to species evolution. Other strains of *Methylobacterium* species, as well as *R. solanacearum* with sbn genes and many taiwachelin-containing strains, were able to rescue Δ*sbnCF* from iron starvation. Thus, strain 22A can utilize these xenosiderophores from *Methylobacterium* species and other non-methylotrophic bacteria. The different types of siderophores and inter-species utilization among *Methylobacterium* species may contribute to the specific species predominance of the genus or even to shape the microbial community in different niches, especially when *Methylobacterium* comprises one of the most predominant species in an environment, such as the phyllosphere.

Among 12 TBDRs in strain 22A, here, we identified two TBDRs for iron uptake (Figure 6), which may enable the strain to more efficiently acquire either siderophore-bound or citrate-bound iron. The *tonB_sbn* is used for the uptake of sbn siderophore. On the other hand, *tonB_cit* is more efficient in iron citrate uptake; however, another TBDR for iron citrate has not yet been identified. The *tonB_Ln* is necessary for Ln-dependent methylotrophy (Figure 6D). The expression of *tonB_Ln* was repressed with increased Ln concentration (Figure 6E). It remains unknown what form of Ln^3+^ is the substrate for *tonB_Ln* (sbn siderophore-bound Ln^3+^ or citrate-Ln^3+^) and whether *tonB_sbn* incorporates sbn siderophore-bound Ln^3+^ or not. The Ln^3+^ concentration-dependent repression of *tonB_Ln*, which was also found in *tonB_LutH* in strain AM1 (Roszczenko-Jasińska et al., 2020), suggested that they have an Ln^3+^-sensing mechanism to optimize Ln^3+^ uptake through TBDR regulation.

*TonB_sbn*, which is homologous to *tonB_Lcc* in strain AM1, was widely conserved among *Methylobacterium* species (Figure 7). The *tonB_Ln* and *tonB_cit* were also well conserved. Interestingly, *tonB_LutH* in strain AM1 was less conserved and found only in clade B (*Methylorubrum* species). The distribution of common or clade specific TBDRs may suggest the possibility of chelator sharing or competition among the *Methylobacterium* species. Other non-characterized TBDRs of strain 22A also showed a wide range of conservation within *Methylobacterium* species; therefore, future exploration of their metal specificity and ionophore will be intriguing.

## Materials and Methods

### Microbial Strains and Culture Conditions

The *Methylobacterium* species used in this study were grown and maintained on an R2A medium or mineral medium containing either 0.5% methanol or 0.5% succinate (herein referred to as methanol and succinate media), as previously described (Alessa et al., 2021). Kanamycin (25 mg/l), rifampicin (20 mg/l), LaCl_3_ (30 μM), La_2_O_3_ (9 mg/l), FeSO_4_ (5–100 μM), and iron citrate (5 or 100 μM) were added when necessary.

The growth tests were conducted in 200 μl various medium prepared in 96-well plates, rotary-shaken at 300 rpm at 28°C. The strains grown on R2A solid media supplemented with iron citrate were harvested by centrifugation and washed with 0.9% NaCl solution to make a cell suspension (OD_600_ = 1.0). The cell suspension was inoculated to a final OD value of 0.02 in the test media condition. Growth was monitored by measuring OD_600_ using a microplate reader (PowerScan HT, Sumitomo Dainippon Pharma, Osaka, Japan).

The wild type strain 22A was grown in methanol media supplemented with 5 μM FeSO_4_ for 5 days. The cells were precipitated at 10,000 × *g* for 10 min and the supernatant was filtered using a 0.2-μm membrane filter. The spent media was stored at –20°C until use. The target strains were grown on 10% wild-type spent media in the appropriate media.

### Detection of Siderophore

Siderophore synthesis was assessed by chrome azurol S (CAS) agar assay for all the strains grown in modified succinate media. In brief, succinate media without Fe^3+^ was mixed with CAS as previously described (Louden et al., 2011). Different methods were used to assess the types of siderophores. Arnow assay for catechol-type (Arnow, 1937): 50 μl of culture supernatant, 50 μl of 0.5 M HCl, and 10 μl reagent (1 g/ml NaNO_2_ and 1 g/ml Na_2_MoO_4_⋅2H_2_O) were mixed. After the formation of the yellow color, 90 μl of 0.1 M NaOH was added, resulting in the generation of red color. 1,2-dihydroxybenzene was used as a standard. Atkin assay for hydroxamate-type (Atkin et al., 1970): 50 μl of culture supernatant was added to 50 μl of the Atkin reagent [0.177 g of Fe(ClO_4_)_3_ and 1.43 g HClO_4_ in 100 ml water]. After 5 min at room temperature, the appearance of a wine-red color indicated the presence of hydroxamate siderophore. Salicylhydroxamic acid was used as a standard. Vogel test for hydroxycarboxylate (Vogel, 1957): the reagent was made by adding 20 μl of phenolphthalein to 60 μl of 1 M NaOH and topping up to 1 ml with water. In total, 50 μl of culture supernatant was mixed with 50 μl of reagent. The disappearance of color by the addition of culture supernatant indicated the presence of hydroxycarboxylate siderophore.

### Construction of Mutant Strains

The primers used in this study are listed in Supplementary Table 4. We generated gene deletion mutants of strain 22A siderophore synthesis gene cluster, Δ*sbnC* (Maq22A_c25435), Δ*sbnF* (Maq22A_c25445), and Δ*sbnCF.* Similarly, TBDR gene deletion mutants, *tonB_cit* (Maq22A_c24885) and *tonB_Ln* (Maq22A_c14845), were also generated in this study. These gene deletion mutants were generated using the allele-replacement vector pK18mobSacB, as previously described. Gene disruption mutants of other *sbn* genes (*sbnA, sbnB, sbnD, sbnG, sbnH*, and Maq22A_c25465) and TBDR genes (*tonB_sbn* (Maq22A_c25420), Maq22A_c06025, Maq22A_c08030, Maq22A_c21870, Maq22A_c22745, Maq22A_c22905, Maq22A_c27600, Maq22A_1p31685, Maq22A_1p34825, and Maq22A_1p38180) were also generated using pK18mobSacB. In brief, the approximately 600 bp mid-portion of the target gene was polymerase chain reaction (PCR)-amplified and cloned into the *Eco*RI site on the pK18mobSacB vector. The vectors were introduced into strain 22A *via* conjugation using *Escherichia coli* S17-1, and the kanamycin-resistant mutants were regarded as the gene disruption mutants. Δ*mxaF* was generated in our previous study (Masuda et al., 2018).

### Construction of Promoter-Reporter Vector

We developed pCM130KmC for general cloning purposes that operates in strain 22A from pCM130 (Addgene plasmid #45828, Marx and Lidstrom, 2001) in our previous study (Yanpirat et al., 2020). A fragment containing an *Eco*RI site and His-tag coding sequence generated by a pair of complementary oligonucleotides (pCM130KmCinsert1 and 2, Supplementary Table 3) was inserted into the *Eco*RI site of pCM130KmC with an In-Fusion Cloning kit to generate pAT01. Next, we also introduced bacterial luciferase (Lux) genes that are PCR-amplified from pUC18-mini-Tn7T-Gm-lux (Wehrmann et al., 2017) with LuxC-F and LuxE-R2 primers into pAT01 to construct a luciferase-reporter vector (pAT06-Lux). The PCR-generated promoter regions of *sbnA*, *tonB_Ln*, *xoxF*, and *mxaF* were cloned into the *Nco*I site on the pAT06-Lux vector. The vectors were introduced into strain 22A *via* conjugation using *E. coli* S17-1 and selected on kanamycin.

To assess the promoter activity, cells of strain 22A and its derivatives transformed with pAT06-Lux containing an appropriate promoter were maintained on R2A supplemented with 25 mg/l Km. The cells were collected, washed with 0.9% NaCl solution, and inoculated into appropriate media (succinate or methanol or methanol/succinate medium) at a final concentration of OD_600_ = 0.02. The culture was replicated in white 96-well plates (for luminescence measurement) and transparent 96-well plates for bacteria growth assessment. The growth conditions were maintained as mentioned above. The maximum luminescence was normalized to cell density (OD_600_) and reported as promoter activity.

### The Physiological Role of the Strain 22A Siderophore

*Methylobacterium* strains grown in R2A were used for the phenotyping assays.

(1) Lanthanum uptake. Strain 22A wild type and Δ*sbnCF* were grown on succinate-methanol media for 5 days, and their spent media was filtered using a 0.2-μm membrane filter. The spent media or varied concentrations of citrate were incubated with La_2_O_3_ 18 mg/l for 24 h at 28°C under shaking at 300 rpm. The sample was centrifuged (10,000 × *g*, 4 min, at 25°C) to remove La_2_O_3_, and 300 μl of the supernatant was dried at 80°C overnight. The dry weight was measured, and the pellets were resuspended in 70% HNO_3_ and boiled at 100°C for 1 h. Samples were diluted to a final concentration of 5% HNO_3_ and analyzed by ICP-MS (Agilent 7500cx). Strain 22A wild type, Δ*sbnCF*, and Δ*mxaF*Δ*sbnCF* were grown in 200 μl methanol medium containing La_2_O_3_/LaCl_3_ and iron citrate in 96-well plates. The plates were shaken at 28°C at 300 rpm and the cell growth was monitored (OD_600_) using a microplate reader.

(2) Oxidative and nitrosative stress resistance. Strain 22A wild type and Δ*sbnCF* were inoculated in liquid R2A media containing 1 mM hydrogen peroxide, 10 mM sodium nitrite, or 10 mM diamide and were incubated at 28°C with continuous shaking at 300 rpm for 24 h in liquid R2A media. The samples were serial diluted and spread on solid R2A media containing 100 μM iron citrate for 48 h. The colony-forming units (CFUs) per milliliter were evaluated, and the percentage of survival in comparison with the unchallenged treatment was calculated.

(3) Heavy metal tolerance. Strain 22A wild type and Δ*sbnCF* were grown in succinate media containing 100 μM of each heavy metal in 96-well plates and their growth was monitored by measuring OD_600_. For cellular metal content quantification, they were grown on succinate in the presence of 25 μM of ZnSO_4_, CuSO_4_, NiSO_4_, and MnSO_4_ for 5 days. The cells were harvested and washed three times with phosphate-buffered saline (PBS) containing 5 mM ethylenediaminetetraacetic acid (EDTA) and then six times with PBS. Bacterial pellets were desiccated at 100°C overnight. Thereafter, the samples were digested and analyzed with the ICP-MS as described above. Iron concentration-dependent heavy metal toxicity was also determined by culturing strain 22A and Δ*sbnCF* in a varied concentration of iron and the heavy metals (50–100 μM).

(4) Biofilm assay. Biofilm formation was studied by a modified method by O’Toole (2011). Strain 22A wild type and Δ*sbnCF* were cultured in succinate media containing 25 to 100 μM FeSO_4_ and iron citrate prepared in 96-well plates at 28°C for 96 h. The content of each well was removed by decantation. The wells were then washed three times with PBS (pH 7.3), air-dried for 45 min, and stained with 0.1% w/v crystal violet solution (200 μl) for 10 min. After three times washing with water, 200 μl of 95% ethanol was added, and the absorbance was determined at 595 nm.

### Interaction of Strain 22A and Plants

(1) Plant growth promotion by strain 22A: Single sterilized duckweed frond was grown in liquid (5 ml) half-strength Murashige and Skoog medium (1/2 MS) under a modified FeSO_4_ concentration of 1 or 10 mg/l. The plants were subjected to inoculation of strain 22A wild type and Δ*sbnCF* at a final concentration of OD_600_ = 0.01 under aseptic conditions. Surface sterilized *A. thaliana* Col-0 seeds were grown on solid 1/2 MS and inoculated with the bacteria as described above. The plants were grown at 23°C with a photoperiod of 16/8 h (light/dark). The experiments were conducted in triplicate. After 28 days, the plants were transferred into separate 1.5-ml tubes and bacterial cells in the media were pelleted at 4,000 × g for 4 min. The plant and bacterial cell fresh weight, dry weight, and metal contents were determined.

(2) *In planta* colonization assay: A rifampicin-resistant spontaneous mutant of strain 22A (strain 22A-rif) and kanamycin-resistant Δ*sbnCF* (Δ*sbnCF*-km) were generated as previously described (Alamgir et al., 2015). *A. thaliana* seeds were inoculated with the 5 μl (OD_600_ = 0.01) of the two strains singly or in combination and grown as described previously. A total of four seedlings were used for each experiment and replicated three times. Then, 5–10 leaves of *A. thaliana* plants not touching the media (200–300 mg fresh weight) were washed in 1.5-ml sterile tubes containing 1 ml 0.9% NaCl. Bacteria were dislodged from the leaf by shaking (5,000 rpm, 10 min) and sonication (34% amplitude, 20 s, Vibra cell, Sonics and Materials Inc.) followed by vortexing for 30 s. The resulting suspensions were serially diluted and plated onto R2A containing rifampicin or kanamycin for CFU determination.

### Characterization of TonB-Dependent Receptor in Strain 22A

A total of 12 TBDRs in strain 22A were assessed for their role in iron-sbn siderophore uptake in 96-well plates. The mutant strains of the 12 TBDR in wild-type backgrounds were grown in succinate media supplemented with 100 μM FeSO_4_. Similarly, the mutants generated under Δ*sbnCF* background were cultivated in succinate media supplemented with 50 μM FeSO_4_ and 10% wild-type spent media, or 100 μM iron citrate, or 50 μM FeSO_4_ and 50 μM citrate. The mutant strains of the 12 TBDR with a background of Δ*mxaF* were also generated to assess the TBDR for Ln uptake.

### Analysis of Siderophore Biosynthesis Clusters and TonB-Dependent Receptors in *Methylobacterium* Species

A search for secondary metabolite biosynthesis genes was performed for the 60 *Methylobacterium* species genome to predict the siderophore gene cluster using AntiSMASH software (version 6.0.1 https://antismash.secondarymetabolites.org) (Blin et al., 2019). Local BLASTp using the siderophore cluster and TBDR genes characterized in strain 22A and (META1p1785 and META1p4129) in strain AM1 as queries was run against the non-redundant protein database of 59 *Methylobacterium* and *Methylorubrum* type strains at threshold values set at ≥ 30% identity.

### Siderophore Cross-Feeding

Δ*sbnCF* cell suspension (OD_600_ = 0.01) in 0.75% agar was overlaid on solid succinate media. A sterilized paper disk (6 mm diameter) was placed on the plate, and the test siderophore donor strains were inoculated onto it. Colony formation of Δ*sbnCF* around the paper disk was checked after 8 days of incubation at 28°C.

### Data Analysis

The statistical analysis (one-way ANOVA followed by Dunnett’s test or Student’s *t*-test) of data was carried out using Prism 6 (GraphPad Software, Inc., CA, United States). Only *p*-values less than a 5% level of significance are shown in the figures.

## Data Availability Statement

The original contributions presented in this study are included in the article/Supplementary Material, further inquiries can be directed to the corresponding author.

## Author Contributions

PJ and YF performed the experiments. PJ, OA, and AT performed the data analysis and drafted the manuscript. All authors designed the research and reviewed the manuscript.

## Conflict of Interest

The authors declare that the research was conducted in the absence of any commercial or financial relationships that could be construed as a potential conflict of interest.

## Publisher’s Note

All claims expressed in this article are solely those of the authors and do not necessarily represent those of their affiliated organizations, or those of the publisher, the editors and the reviewers. Any product that may be evaluated in this article, or claim that may be made by its manufacturer, is not guaranteed or endorsed by the publisher.
