# Supplementary material for: Siderophore for Lanthanide and Iron Uptake for Methylotrophy and Plant Growth Promotion in Methylobacterium aquaticum Strain 22A
Source: Front Microbiol. 2022 Jul 7;13:921635. doi: 10.3389/fmicb.2022.921635 (PMC9301485; doi:10.3389/fmicb.2022.921635)
Supplement: Supplementary file 1 [file Data_Sheet_1.PDF]

Supplementary file
Patrick Juma et al.

Siderophore for lanthanide and iron uptake for methylotrophy and plant growth promotion in *Methylobacterium aquaticum* strain 22A

Patrick Juma, Yoshiko Fujitani, Ola Alessa, Tokitaka Oyama, Hiroya Yurimoto, Yasuyoshi Sakai, and Akio Tani

Fig. S1. Gene organization of sbn siderophore cluster in different *Methylobacterium* species, *R. solanacearum, C. metallidurans,* and *S. aureus,* visualized by Clinker (Gilchrist and Chooi 2021).

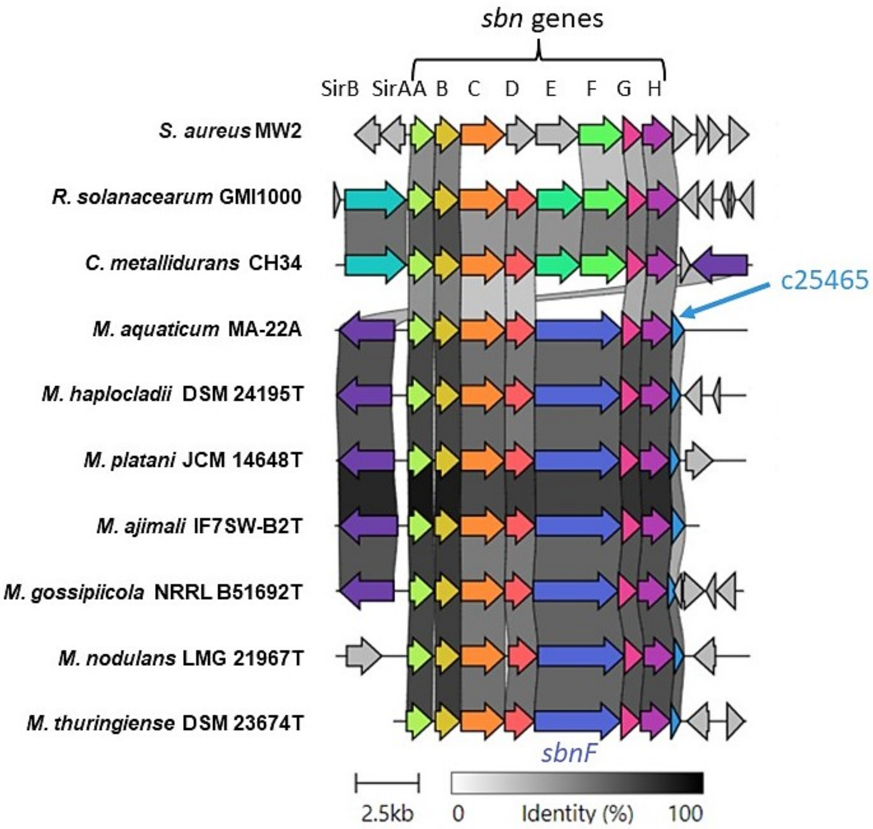

Supplementary file
Patrick Juma et al.

Fig. S2. A. Assay for different types of siderophores using the spent medium of *M. aquaticum* strain 22A grown on succinate for 7 days. 1,2-dihydroxybenzene and salicylhydroxamate (both 10 g/l) were used as positive controls for Arnow assay and Atkin assay, respectively. B. Siderophore production assay of each *sbn* gene mutant and wild-type strain 22A on CAS plate.

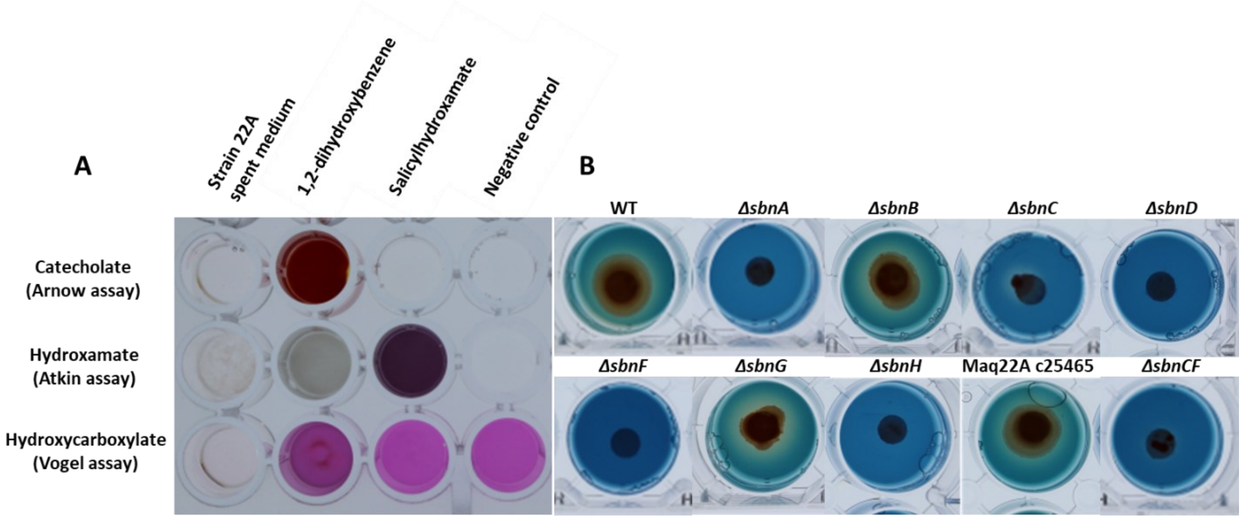

Fig. S3. A. *Arabidopsis thaliana* growth in 1/2 MS media inoculated with none (non-inoculated control, NIC), strain 22A wild type (WT), and Δ*sbnCF* under 10 and 1 mg/l $FeSO_4$. B. Phyllosphere colonization of strain 22A wild type (WT) and Δ*sbnCF* singly inoculated or co-inoculated on *A. thaliana* under 1 mg/l $FeSO_4$. Data represent the mean of three replicates ± standard deviation (SD).

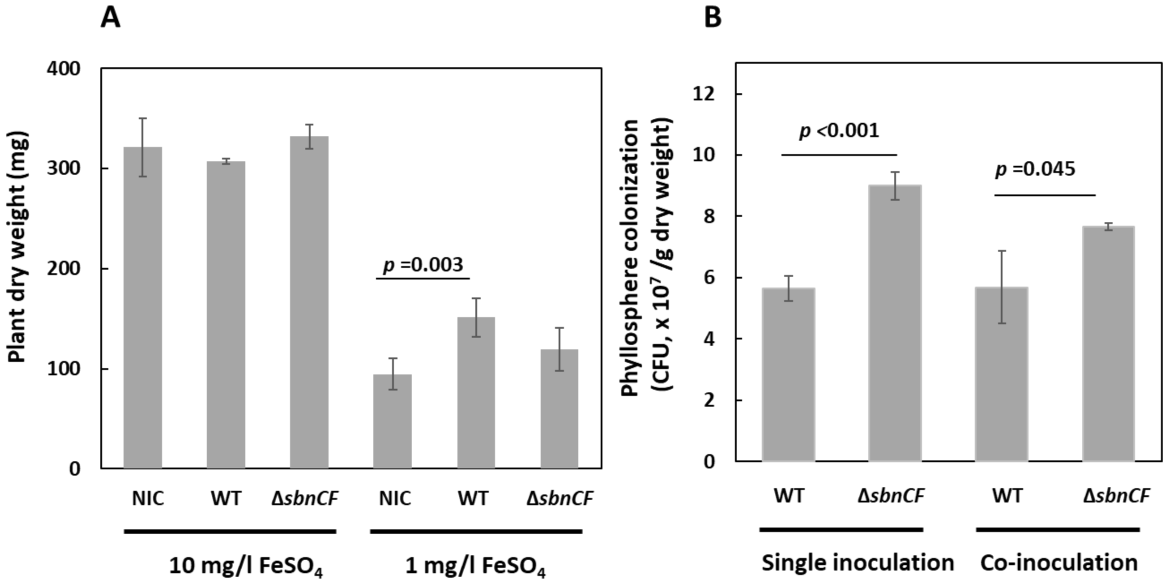

Supplementary file
Patrick Juma et al.

Fig. S4. A. Biofilm formation of the wild-type strain 22A and Δ*sbnCF* under different concentrations of $FeSO_4$ or iron citrate. Bars, biofilm formation (evaluated by A_{595}/OD_{600}); circles, cell yield (OD_{600}). B. Survival of the wild-type strain 22A and Δ*sbnCF* after treatment of H_2O_2, diamide, and $NaNO_2$. Data represent the mean of three replicates ± standard deviation (SD).

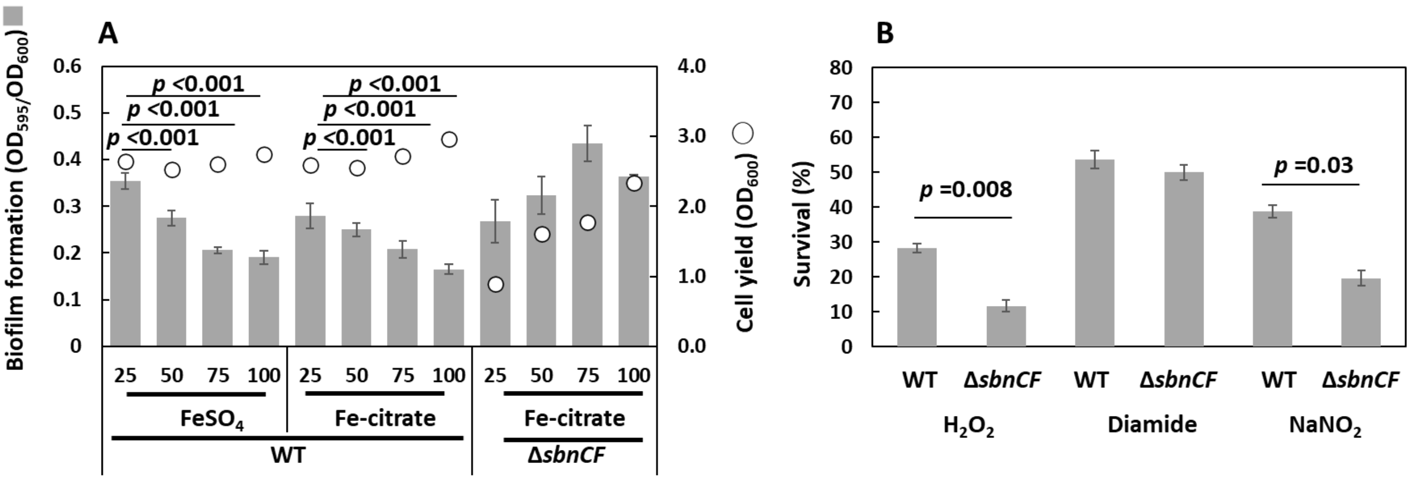

Reference

Gilchrist, C. L. M. Chooi, Y.-H. Clinker & clustermap.js: Automatic generation of gene cluster comparison figures. Bioinformatics (2021) https:/doi.org/10.1093/bioinformatics/btab007.
